# Affected Persons in Laboratory Exposures to Human Pathogens and Toxins in Canada, 2016–2024: A Sector‐Specific Analysis

**DOI:** 10.1002/ajim.70137

**Published:** 2026-09-15

**Authors:** Emily F. Tran, Christine Abalos, Antoinette N. Davis, Audrey Gauthier, Samuel Bonti‐Ankomah

**Affiliations:** ^1^ Regulatory, Operations and Emergency Management Branch, Public Health Agency of Canada Ottawa Ontario Canada

**Keywords:** affected persons, human pathogens and toxins, laboratory exposure incidents, Laboratory Incident Notification Canada, laboratory‐acquired infections, sector

## Abstract

**Background:**

Exposures to human pathogens and toxins in licensed facilities in Canada have been monitored by a federal surveillance system since 2015, yet the affected persons (APs) in these incidents remain uncharacterized. This study comprehensively describes APs, highlighting sector‐specific patterns and trends over time.

**Methods:**

Public Health Agency of Canada data on APs in exposure incidents between 2016 and 2024 (*n* = 1058) were analyzed. APs were described by disease outcome (APs who developed a laboratory‐acquired infection [LAI]; APs who did not develop an LAI), role, sector, education, years of experience, route of exposure, main activity during exposure, exposure severity, root cause of exposure, occurrence type of exposure, post‐exposure interventions and implicated agents.

**Results:**

The majority of APs were in the non‐LAI group (96.6%), were technicians/technologists (74.5%), worked in the hospital sector (56.3%), completed a technical/trades college diploma (52.8%), were exposed via inhalation (62.7%), were performing microbiology activities during exposure (75.0%) and sought occupational health consultation within seven days of exposure (61.4%). The distributions of role, education, and route of exposure were most heterogeneous in the academic, other government and veterinary/animal health sectors and least heterogeneous in the hospital and public health sectors.

**Conclusions:**

Distributions of AP‐related variables differed across sectors, varying most in the academic, other government, and veterinary/animal health sectors. Study findings contribute to the characterization of APs involved in exposure incidents and may inform future research aimed at identifying factors associated with exposure incidents and LAIs.

## 1 Introduction

Personnel working with human pathogens or toxins (HPTs) may be in close proximity to materials that have the potential to cause infection or intoxication. This risk of exposure exists while conducting activities such as microbiological work, in vivo animal research, cell culture, and microscopy, among others [1]. Exposures may occur through various transmission routes, including, but not limited to, inhalation, inoculation or injection through needle, sharps, bite or scratch, or absorption via contact with mucous membranes or skin [2, 3], potentially leading to a laboratory‐acquired infection (LAI) [4, 5]. LAIs are not only a concern for the individual, but also for the greater community, as transmission could extend beyond the facility or containment zone. For instance, in 2009, an exposure incident involving *Yersinia pestis* (bacterium that causes the plague) resulted in the LAI‐related death of a researcher and the administration of antimicrobial prophylaxis to 30 other laboratory members, one household contact, and 61 other close contacts in the United States [6].

Most studies examining HPT exposure incidents on a global scale have primarily focused on LAIs as well as trends in pathogen types, root causes of incidents, and laboratory activities at the time of exposure [5, 7, 8]. However, analyses of the individuals affected in these incidents are minimal [5] or lacking [7, 8]. The main observations of individuals affected in LAIs worldwide indicate that the majority were laboratory technicians infected via airborne transmission or inhalation [5, 9] and were prescribed post‐exposure treatment [5]. These findings were similarly reported in a study describing LAIs in Canada from 2016 to 2021 [10]. One of the few studies to include demographic data—a review of LAIs specifically associated with brucellosis in China—found that younger individuals, females, and students were predominantly affected [11]. Overall, the literature on affected persons (APs) in exposure incidents is limited. Particularly, there is a knowledge gap regarding APs who did not develop an LAI, which, based on a study using data from licensed Canadian facilities, account for the majority of exposed individuals [12].

In Canada, all facilities where the handling of HPTs of risk group (RG) 2 or higher occur are required to obtain a licence under the *Human Pathogens and Toxins Act* (HPTA) and must report incidents to the Minister of Health through the Public Health Agency of Canada (PHAC) [13]. The information reported includes details on the APs in non‐LAI incidents and LAI‐associated incidents. The reporting of these incidents is captured through a federal surveillance system, Laboratory Incident Notification Canada (LINC). Although previous LINC studies assessing exposure incidents to HPTs in facilities in Canada did include APs from non‐LAI incidents [1, 2, 3, 12, 14, 15, 16, 17, 18], the analyses were brief and only provided an overview of a few factors such as their role, years of experience, route of exposure, and post‐exposure intervention. A more extensive examination of APs in exposure incidents could contribute to improved preventive measures, practices, and policies, thus improving the health and safety of personnel and the greater population. Specifically, the differences in APs between sectors should be investigated as past annual reports by LINC have found sector‐specific variations in exposure incidents [16, 18]. Aside from Canada, there are few studies at the national level examining the characteristics of individuals affected in non‐LAI incidents to HPTs.

The objective of this study is to comprehensively describe persons affected in exposure incidents in licensed facilities in Canada using LINC data for incidents that occurred between January 1, 2016, and December 31, 2024, highlighting sector‐specific patterns and trends over time.

## 2 Materials and Methods

### 2.1 Data Source and Collection

The LINC surveillance system captures incident reports from facilities in Canada that are regulated by PHAC. Facilities use the Biosecurity Portal, a customized platform through Microsoft Dynamics Customer Relations Management system, to submit standardized forms for incidents involving HPTs. Many data fields are mandatory, allowing for specificity and uniformity in the reported data. For every exposure report submitted, one or more follow‐up reports may be submitted. Data on all APs in an exposure incident are collected through the follow‐up reports. Only information from the most recently submitted follow‐up report was retained for this study. This project constituted public health surveillance rather than human subjects research; therefore, review by an institutional research ethics board was not required. No identifiable personal information was collected, and informed consent was not required.

### 2.2 Inclusion Criteria

Following the receipt of reports, accuracy and completeness of answers is validated by PHAC. Exposure incidents that were deemed to be outside the scope of the HPTA were categorized as “ruled out” and excluded from this study. Such reports included incidents involving RG1 pathogens, pathogens in their natural environment that were not isolated, or cases in which further investigation concluded there was indeed no exposure. APs where the exposure outcome was ruled out were also excluded from the analysis.

### 2.3 Study Variables

Exposure incidents were described by sector, implicated HPTs, main activity performed during the incident, type of exposure incident (i.e., LAI‐associated incident vs. non‐LAI incident), occurrence type, root cause of the incident, route of exposure, and exposure severity. Incidents in which at least one AP developed a suspected or confirmed LAI were classified as LAI‐associated incidents, while the remaining incidents were classified as non‐LAI incidents. Licensed facilities were associated with one of the following sectors: academic, environmental health—government (environmental health), hospital, other government, public health—government (public health), private industry/business, or veterinary/animal health—government (veterinary/animal health). Definitions of the main activities, occurrence types, and exposure severities are provided in Tables [Link], [Link], [Link], respectively, and examples of areas of concern for each root cause type are provided in Table S4. Facilities may cite multiple occurrence types and root causes for an exposure incident.

More than one AP could be associated with an incident. AP‐level variables included disease outcome, role, highest level of education, years of laboratory experience, and the interventions employed post‐exposure. Disease outcome categories included exposure, ruled out, seroconversion, suspected LAI, and confirmed LAI. APs who developed an LAI, including seroconversion or a suspected or confirmed LAI were classified as the LAI group, whereas APs who did not develop an LAI were classified as the non‐LAI group. When multiple APs were associated with the same incident, APs were classified individually according to their disease outcome, regardless of the incident classification. Other organizational data such as work hours and workforce size, and demographic data are not collected by LINC.

### 2.4 Analysis

The data extract included the follow‐up reports of exposure incidents that occurred from January 1, 2016, to December 31, 2024, as well as information on individuals affected in those exposure incidents. Descriptive statistics for continuous and categorical variables were obtained. To compare the distribution of APs in AP‐related variables (e.g., role, highest education level, route of exposure, post‐exposure interventions) between groups (i.e., APs in the LAI group vs. the non‐LAI group, APs across sectors), Welch's *t*‐tests/ANOVA or Kruskal‐Wallis tests were used for continuous variables and chi‐square or Fisher's tests were used for categorical variables. Effect sizes were calculated using Cohen's *d* or ordinal eta‐squared (η^2^_H_) for continuous variables and Cramér's V for categorical variables to assess the magnitude of observed differences. As an exploratory analysis, Firth's penalized logistic regression was used to assess potential associations between selected AP and exposure characteristics and the development of an LAI. This approach was chosen to account for sparse data and the small number of APs in the LAI group. Statistical significance was defined as a p‐value less than 0.05. Although the nature of this study was descriptive, a False Discovery Rate (FDR) corrected p‐value was also considered to address potential false positives due to multiple comparisons for sector‐specific analyses.

Analyses were conducted in R 4.4.2, and Microsoft Excel was used to validate data.

## 3 Results

There were 1058 people affected in incidents in licensed facilities in Canada between January 1, 2016, and December 31, 2024. Of the affected individuals, 3.4% were in the LAI group and 96.6% were in the non‐LAI group. The characteristics of the APs by disease outcome (LAI group vs. non‐LAI group) are presented in Table 1. Most APs were technicians/technologists (74.5%), worked in the hospital sector (56.3%), completed a technical/trades college diploma (52.8%), were exposed via inhalation (62.7%), were performing microbiology activities at the time of the exposure incident (75.0%), and were involved in an exposure of minor severity (54.0%).

**Table 1:** Characteristics of affected persons by disease outcome (LAI group vs. non‐LAI group), 2016–2024 (*N* = 1058).

	LAI group (*n* = 36; 3.4%)		Non‐LAI group (*n* = 1022; 96.6%)		Total (*N* = 1058)		*p*‐value	Effect size^a^
	n	%	n	%	n	%		
**Role**							0.158	0.04
Animal handler	0	0	12	1.2	12	1.1		
Researcher	2	5.6	31	3.0	33	3.1		
Student	8	22.2	105	10.3	113	10.7		
Supervisor/Manager	1	2.8	19	1.9	20	1.9		
Technician/technologist	21	58.3	767	75.0	788	74.5		
Other	4	11.1	78	7.6	82	7.8		
Unknown	0	0	10	1.0	10	0.9		
**Sector**							0.006	0.10
Academic	13	36.1	180	17.6	193	18.2		
Environmental Health – Government	0	0	2	0.2	2	0.2		
Hospital	11	30.6	585	57.2	596	56.3		
Other Government	1	2.8	18	1.8	19	1.8		
Private Industry/Business	4	11.1	96	9.4	100	9.5		
Public Health – Government	4	11.1	120	11.7	124	11.7		
Veterinary/Animal Health – Government	3	8.3	21	2.1	24	2.3		
**Highest education level**							< 0.001	0.20
Grade School	1	2.8	0	0	1	0.1		
High School	3	8.3	58	5.7	61	5.8		
Technical/Trades college diploma	15	41.7	544	53.2	559	52.8		
Bachelor's degree	11	30.6	230	22.5	241	22.8		
Master's degree	3	8.3	65	6.4	68	6.4		
MD/PhD	0	0	38	3.7	38	3.6		
Postdoctoral fellowship	2	5.6	4	0.4	6	0.6		
Other	0	0	13	1.3	13	1.2		
Unknown	1	2.8	70	6.8	71	6.7		
**Years of laboratory experience**								
Mean (SD)	8.1 (9.9)		11.4 (10.7)		11.3 (10.7)		0.066	−0.32
Median (IQR)	4.5 (8.0)		8.0 (15.0)		7.5 (16.0)		0.039	0.003
**Route of exposure**							< 0.001	0.35
Absorption via contact with mucous membrane contact	1	2.8	26	2.5	27	2.6		
Absorption via contact with skin	2	5.6	43	4.2	45	4.3		
Ingestion	7	19.4	5	0.5	12	1.1		
Inhalation	6	16.7	657	64.3	663	62.7		
Inoculation/injection – bite/scratch	0	0	21	2.1	21	2.0		
Inoculation/injection – needle/sharps	6	16.7	127	12.4	133	12.6		
Other	3	8.3	42	4.1	45	4.3		
Unknown	11	30.6	101	9.9	112	10.6		
**Main activity**							< 0.001	0.25
Animal care	1	2.8	12	1.2	13	1.2		
Autopsy/necropsy	3	8.3	18	1.8	21	2.0		
Cell culture	0	0	28	2.7	28	2.6		
Education/training	0	0	7	0.7	7	0.7		
*In vivo* animal research	5	13.9	71	6.9	76	7.2		
Maintenance	1	2.8	14	1.4	15	1.4		
Microbiology	18	50.0	775	75.8	793	75.0		
Microscopy	0	0	10	1.0	10	0.9		
Molecular investigations	0	0	18	1.8	18	1.7		
Serology/hematology	0	0	4	0.4	4	0.4		
Other	6	16.7	65	6.4	71	6.7		
Unknown	2	5.6	0	0	2	0.2		
**Exposure severity**							< 0.001	0.27
Negligible	4	11.1	288	28.2	292	27.6		
Minor	9	25.0	562	55.0	571	54.0		
Moderate	19	52.8	159	15.6	178	16.8		
Major	2	5.6	1	0.1	3	0.3		
Unknown	2	5.6	12	1.2	14	1.3		

Abbreviations: IQR, interquartile range; SD, standard deviation.

^a^ Cramér's V for categorical variables, Cohen's *d* or η^2^_H_ for continuous variables.

The distribution of APs differed between the LAI group and the non‐LAI group for all the characteristics in Table 1 except for role (*p* = 0.158) and the mean number of years of laboratory experience (*p* = 0.066). The mean years of experience differed modestly between APs in the LAI group and the non‐LAI group, with the LAI group reporting fewer years of experience than the non‐LAI group (Cohen's *d* = −0.32), indicating a small effect. The difference in median years of experience corresponded to a negligible effect size (η^2^_H_ = 0.003), suggesting limited practical significance. While the two most common roles of APs in both the LAI and non‐LAI groups were technician/technologist and student, there was a higher proportion of students, researchers, supervisors, and APs that held an “other” role in the LAI group. Compared to APs in the non‐LAI group, those in the LAI group were more likely to be working in the academic and veterinary/animal health sectors, have completed a bachelor's degree or postdoctoral fellowship, have fewer median years of laboratory experience, be exposed via unknown pathways or ingestion, be performing in vivo animal research or autopsy/necropsy activities at the time of exposure, and be involved in exposures of higher (i.e., moderate or major) severity. APs in the non‐LAI group were more likely to be working in the hospital sector, have a technical/trades college diploma, be exposed via inhalation, be performing microbiology activities during exposure, and be involved in an exposure of lower (i.e., negligible or minor) severity. When evaluating effect size in Table 1, estimates indicated that associations between the variables examined and the development of an LAI were generally weak to moderate (Cramér's V = 0.04–0.35), with route of exposure having the highest effect size. This suggests that while certain factors differed between those in the LAI group and the non‐LAI group, the practical magnitude of these differences was limited. In addition, exploratory analysis using Firth's penalized logistic regression comparing APs in the LAI group with those in the non‐LAI group identified potential associations (data not shown). The strongest observed association was for APs involved in exposure incidents classified as moderate in severity, which were more likely to be associated with the development of an LAI (OR = 30.7, 95% CI: 6.0–331.5). Other statistically significant associations were observed for AP sector, route of exposure, and main activity. However, several estimates were imprecise and had wide confidence intervals, reflecting the small number of APs in the LAI group and sparse subgroup counts. As such, these findings should be considered exploratory and hypothesis‐generating.

Of the 10 possible exposure occurrence types, procedure‐related occurrences were the most commonly associated with APs (49.4%) (Table S5). However, APs in the LAI group were less likely to be involved in procedure‐related occurrences (*p* = 0.003) and more likely to be involved in an unknown occurrence type (*p* < 0.001), compared to APs in the non‐LAI group. Those in the LAI group were also more likely to be involved in an exposure citing the “unknown” category for most of the possible root causes of an incident (Table S6).

### 3.1 Highest Level of Education Versus Years of Experience

The association between highest education level and years of experience of APs was evaluated since both variables represent the extent of an individual's knowledge and training. The two variables were significantly associated (*p* < 0.001) (data not shown). Individuals with a technical/trades college diploma had the highest median years of experience (median = 10.0, interquartile range [IQR] = 16.0), followed by those with an MD/PhD (median = 9.0, IQR = 10.5). Excluding the one AP with grade school (grades 1–8) as the highest level of education (years of experience = 0), the lowest years of experience were among APs with a high school diploma (median=1.0, IQR = 9.0) as the highest level of education.

### 3.2 Post‐Exposure Interventions

Post‐exposure interventions included first aid, medical consultation, occupational health consultation, drug treatment, and post‐exposure prophylaxis (PEP). There were two (5.6%) APs in the LAI group and 125 (12.2%) APs in the non‐LAI group that did not seek any post‐exposure intervention. On average, APs in the LAI group received slightly more post‐exposure interventions (mean ± standard deviation [SD] = 2.58 ± 1.52) than APs in the non‐LAI group (mean ± SD = 2.13 ± 1.37) (*p* = 0.085). The most frequently implemented interventions overall were occupational health consultation within seven days (61.4%) and medical consultation within seven days (47.8%) (Table 2). Compared to APs in the non‐LAI group, those in the LAI group were more likely to seek medical consultation more than seven days after exposure (*p* < 0.001) or drug treatment more than seven days after exposure (*p* < 0.001), and were less likely to seek occupational health consultation within seven days after exposure (*p* = 0.001) or PEP within seven days (*p* = 0.015). The strength of association between the type of exposure incident and post‐exposure interventions administered to APs was generally negligible to weak, with Cramér's V values ranging from 0.00 to 0.24.

**Table 2:** Post‐exposure interventions administered to affected persons by disease outcome (LAI group vs. non‐LAI group), 2016–2024 (*N* = 1058).

	LAI group (*n* = 36; 3.4%)		Non‐LAI group (*n* = 1022; 96.6%)		Total (*N* = 1058)		*p*‐value	Effect size
	n	%	n	%	n	%		
**First‐aid administered immediately after exposure**							0.823	0
Yes	7	19.4	178	17.4	185	17.5		
No	29	80.6	844	82.6	873	82.5		
**Medical consultation within 7 days**							0.127	0.050
Yes	22	61.1	484	47.4	506	47.8		
No	14	38.9	538	52.6	552	52.2		
**Medical consultation more than 7 days after the exposure**							< 0.001	0.167
Yes	21	58.3	204	20.0	225	21.3		
No	15	41.7	818	80.0	833	78.7		
**Occupational health consultation within 7 days**							0.001	0.104
Yes	12	33.3	638	62.4	650	61.4		
No	24	66.7	384	37.6	408	38.6		
**Occupational health consultation more than 7 days after exposure**							0.231	0.027
Yes	12	33.3	242	23.7	254	24.0		
No	24	66.7	780	76.3	804	76.0		
**Drug treatment more than 7 days after the exposure**							< 0.001	0.243
Yes	13	36.1	48	4.7	61	5.8		
No	23	63.9	974	95.3	997	94.2		
**PEP within 7 days**							0.015	0.067
Yes	4	11.1	302	29.5	306	28.9		
No	32	88.9	720	70.5	752	71.1		
**PEP more than 7 days after exposure**							1.00	0
Yes	2	5.6	81	7.9	83	7.8		
No	34	94.4	941	92.1	975	92.2		

Abbreviation: PEP, post‐exposure prophylaxis.

### 3.3 Sector‐Specific Patterns

#### 3.3.1 Characteristics of Affected Persons

Before adjusting for multiple comparisons, the sector in which APs were working was statistically significantly associated with role, highest education level, years of experience, route of exposure, main activity during exposure, exposure severity, all occurrence types except for insect‐related, and all root causes (*p* < 0.05) (data not shown). These associations remained statistically significant even after the FDR correction. The role, highest education level, and route of exposure of APs varied most greatly in the academic, other government and veterinary/animal health sectors (Figure 1). The other government sector includes facilities working with HPTs at the federal, provincial/territorial, and municipal levels that are not classified under environmental health, public health, or veterinary/animal health.

**Figure 1:**
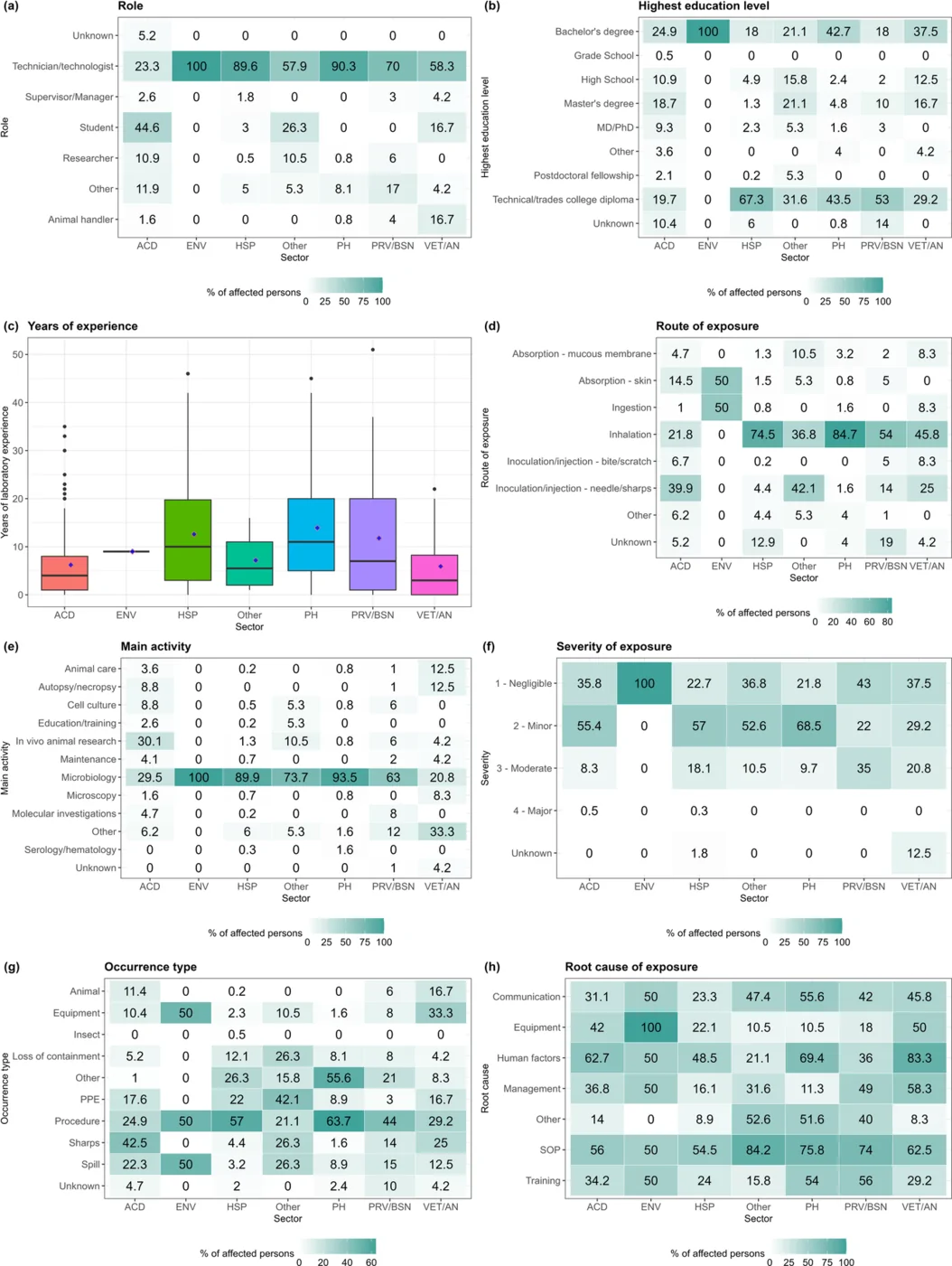
Distribution of affected persons characteristics across sectors, 2016–2024. The sectors are represented on the x‐axis, and the following characteristics are represented on the y‐axis: (a) Role, (b) Highest education level, (c) Years of laboratory experience, (d) Route of exposure, (e) Main activity during exposure, (f) Severity of the exposure, (g) Occurrence type and (h) Root cause of the exposure. In plot (c), the blue diamond corresponds to the mean years of laboratory experience in each sector, and the black dots represent the outliers. For plots (a), (b), (d), (e), (f), (g) and (h), each tile represents the proportion of affected persons in each sector that was categorized into the corresponding y‐axis category. Abbreviations: ACD (Academic); ENV (Environmental Health – Government); HSP (Hospital); Other (Other Government); PH (Public Health – Government); PRV/BSN (Private Industry/Business); VET/AN (Veterinary/Animal Health – Government); PPE (personal protective equipment); SOP (standard operating procedure).

The academic sector was the only sector with APs in every role. All other sectors lacked APs in at least one of the roles (Figure 1a). Most APs in the academic sector were students (44.6%) and technicians/technologists (23.3%), while those in the other sectors were predominantly technicians/technologists (57.9%–100%).

The highest education level of APs was most commonly a bachelor's degree in the academic, environmental health, and veterinary/animal health sectors (24.9%–100%), whereas in the hospital, other government, public health, and private industry/business sectors, it was a technical/trades college diploma (31.6%–67.3%) (Figure 1b).

APs in the academic, other government, and veterinary/animal health sectors had the fewest years of laboratory experience (mean = 5.9–7.2, median = 3.0–5.5), while those in the public health sector had the most years of experience (mean = 13.9, median = 11.0) (Figure 1c). The range was greatest in the hospital (IQR = 16.8) and private industry/business (IQR = 19.0) sectors.

The predominant route of exposure differed by sector. While inhalation was the leading route of exposure for hospital, public health and veterinary/animal health sectors (45.8%–84.7%) (Figure 1d), APs in the academic and other government sectors were most frequently exposed via inoculation/injections from needles/sharps (39.9% and 42.1%, respectively), and in the environmental health sector, the most frequent route of exposure was evenly split between skin absorption and ingestion.

Microbiology‐related activities accounted for the majority of activities performed at the time of the exposure incident in all sectors (63.0%–100%), with the exception of the academic and veterinary/animal health sectors (Figure 1e). In the academic sector, APs conducted in vivo animal research activities (30.1%) slightly more often than microbiology (29.5%) at the time of exposure. The most common activity in the veterinary/animal health sector was “other” (33.3%), followed by microbiology (20.8%). “Other” included activities such as microtomy and histology.

More than half of the APs were involved in exposure incidents of minor severity in the academic (55.4%), hospital (57.0%), other government (52.6%), and public health (68.5%) sectors (Figure 1f). APs in the remaining sectors were most frequently involved in exposure incidents of negligible severity (37.5%–100%). The private industry/business sector contributed the largest proportion of APs involved in exposure incidents of moderate severity (35.0%).

APs in the hospital, public health and private industry/business sectors were most frequently involved in procedure‐related occurrences (44.0%–63.7%) (Figure 1g). In the academic, other government, and veterinary/animal health sectors, APs were most commonly involved in sharps‐related occurrences (42.5%), PPE‐related occurrences (42.1%) and equipment‐related occurrences (33.3%), respectively. Half of the APs in the environmental health sector were involved in each of the following occurrences: equipment‐, procedure‐, and spill‐related.

The most commonly cited root cause of exposure in the hospital, other government, public health, and private industry/business sectors was standard operating procedure (SOP)‐related (54.5%–84.2%) (Figure 1h). APs in the academic and veterinary/health sectors were most frequently involved in exposure incidents that cited human factors as a root cause (62.7% and 83.3%, respectively), while APs in the environmental health sector most frequently cited equipment as a root cause (100%).

#### 3.3.2 Post‐Exposure Interventions

Aside from the environmental health sector, where all APs did not seek any post‐exposure intervention, the veterinary/animal health and private industry/business sectors had the highest proportion of APs who did not seek any post‐exposure intervention (33.3% and 25.0%, respectively), while the public health sector had the lowest proportion of APs that did not seek any post‐exposure intervention (4.8%). The mean number of post‐exposure interventions per AP was highest in the public health and private industry/business sectors (mean ± SD: 2.66 ± 1.00 and 2.37 ± 1.85, respectively) and, excluding the environmental health sector, was lowest in the veterinary/animal health and academic sectors (mean ± SD: 1.38 ± 1.38 and 1.69 ± 1.43, respectively) (*p* < 0.001). The distribution of APs in all of the post‐exposure interventions differed significantly by sector (*p* < 0.001), except for administration of drug treatment more than seven days after the exposure (*p* = 0.456), which was not statistically significant before and after FDR correction for multiple testing (data not shown). The most common intervention in the academic and veterinary/animal health sectors was administration of first‐aid immediately after exposure (58.0% and 41.7%, respectively) (Figure 2). Occupational health consultation within seven days after exposure occurred most commonly in the hospital (71.6%) and public health (81.5%) sectors. More than half of the APs in the public health sector also sought medical consultation within seven days of exposure (76.6%) and occupational health consultation more than seven days after exposure (54.8%). In the other government sector, medical consultation within seven days of exposure was the most predominant intervention among APs (63.2%). Two interventions in the private industry/business sector were employed by relatively similar proportions of APs: medical consultation within seven days (58.0%) and occupational health consultation within seven days (54.0%).

**Figure 2:**
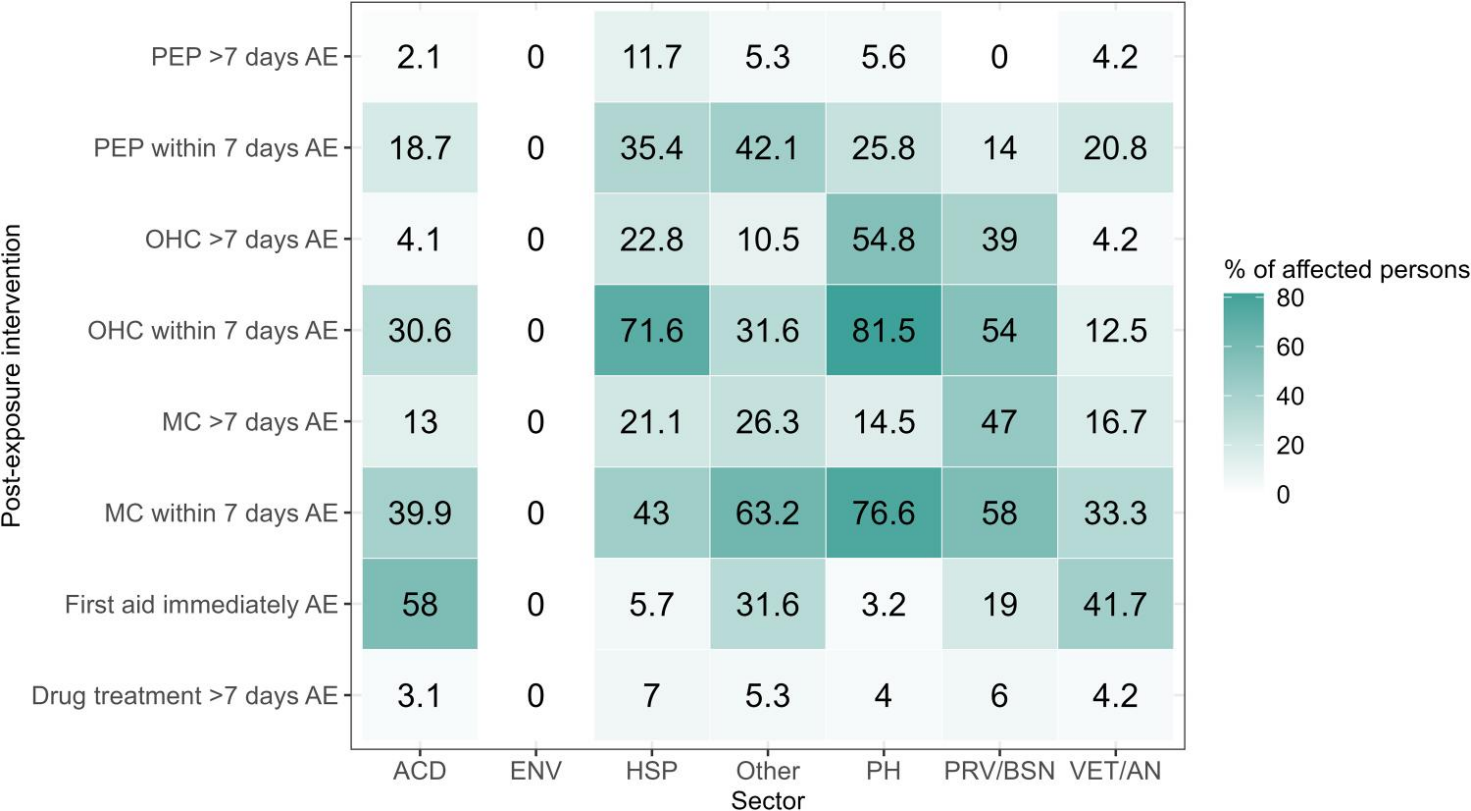
Post‐exposure interventions administered to affected persons by sector, 2016–2024. The post‐exposure interventions are represented on the y‐axis and the sectors are represented on the x‐axis. Each tile represents the proportion of affected persons in each sector that employed the specific intervention. ACD (Academic); ENV (Environmental Health – Government); HSP (Hospital); Other (Other Government); PH (Public Health – Government); PRV/BSN (Private Industry/Business); VET/AN (Veterinary/Animal Health – Government); AE (after exposure); MC (medical consultation); OHC (occupational health consultation); PEP (post‐exposure prophylaxis).

### 3.4 Implicated Biological Agents

The most commonly implicated agents based on the proportion of APs in each sector are shown in Table 3. Overall, bacteria were the most common agent type in exposure incidents. APs were most frequently affected by *Staphylococcus aureus* in the academic sector (9.8%), *Brucella melitensis* in the hospital (34.1%) and public health sectors (39.5%), *Burkholderia pseudomallei* in the other government sector (31.6%), *Brucella suis* in the private industry/business sector (29.0%), and chronic wasting disease agent in the veterinary/animal health sector (45.8%). At least one species from the *Brucella* genus was among the three most commonly implicated agents in the academic, hospital, public health, and private industry/business sectors. At least one of the most implicated agents in every sector was an RG3 HPT, while all three of the most commonly implicated agents in the hospital and private industry/business sectors were classified as RG3.

**Table 3:** Three most common biological agents by proportion of affected persons in each sector and overall, 2016–2024.

Sector	Agent name	Risk group	Agent type	% of affected persons
Academic (*n* = 193)	*Staphylococcus aureus*	RG2	Bacteria	9.8
	Severe acute respiratory syndrome coronavirus 2 (SARS‐CoV‐2)	RG3	Virus	5.7
	*Brucella ovis*	RG3	Bacteria	5.2
Environmental Health – Government (*n* = 2)	*Enterococcus spp*.	RG2	Bacteria	50.0
	*Escherichia coli* (Migula) Castellani and Chalmers (ATCC 25922)	RG2	Bacteria	50.0
Hospital (*n* = 596)	*Brucella melitensis*	RG3	Bacteria	34.1
	*Brucella abortus*	RG3	Bacteria	11.1
	*Coccidioides immitis*	RG3	Fungus	9.1
Other Government (*n* = 19)	*Burkholderia pseudomallei*	RG3	Bacteria	31.6
	*Escherichia coli enterohemorrhagic* ‐ VTEC (EHEC)	RG2	Bacteria	15.8
	*Pseudomonas aeruginosa*	RG2	Bacteria	10.5
	*Staphylococcus aureus*	RG2	Bacteria	10.5
Private Industry/Business (*n* = 100)	*Brucella suis*	RG3	Bacteria	29.0
	*Burkholderia pseudomallei*	RG3	Bacteria	9.0
	Human immunodeficiency virus 1 and 2	RG3	Virus	8.0
Public Health – Government (*n* = 124)	*Brucella melitensis*	RG3	Bacteria	39.5
	*Neisseria meningitidis*	RG2	Bacteria	17.7
	*Francisella tularensis*	RG3	Bacteria	14.5
Veterinary/Animal Health – Government (*n* = 24)	Chronic wasting disease agent	RG2	Prion	41.7
	Scrapie agent	RG2	Prion	25.0
	Bovine spongiform encephalopathy agent	RG3	Prion	12.5
Overall (*N* = 1058)^a^	*Brucella melitensis*	RG3	Bacteria	24.3
	*Brucella abortus*	RG3	Bacteria	6.2
	*Neisseria meningitidis*	RG2	Bacteria	5.8
	*Francisella tularensis*	RG3	Bacteria	5.8

^a^ Four biological agents are shown in the Overall section due to a tie for the third‐highest proportion.

### 3.5 Time Trends

The mean number of APs per exposure incident from 2016 to 2024 remained consistent, averaging 2.05 (SD = 0.53) APs per incident. Annual means fluctuated, with a slightly decreasing trend despite peaks in 2018 (mean = 2.77) and 2022 (mean = 2.58) (Figure 3a). The mean number of APs per active licence, however, did not fluctuate between 2016 and 2024 (mean ± SD = 0.12 ± 0.06) except for a peak in 2018 (mean = 0.27) (Figure 3b).

**Figure 3:**
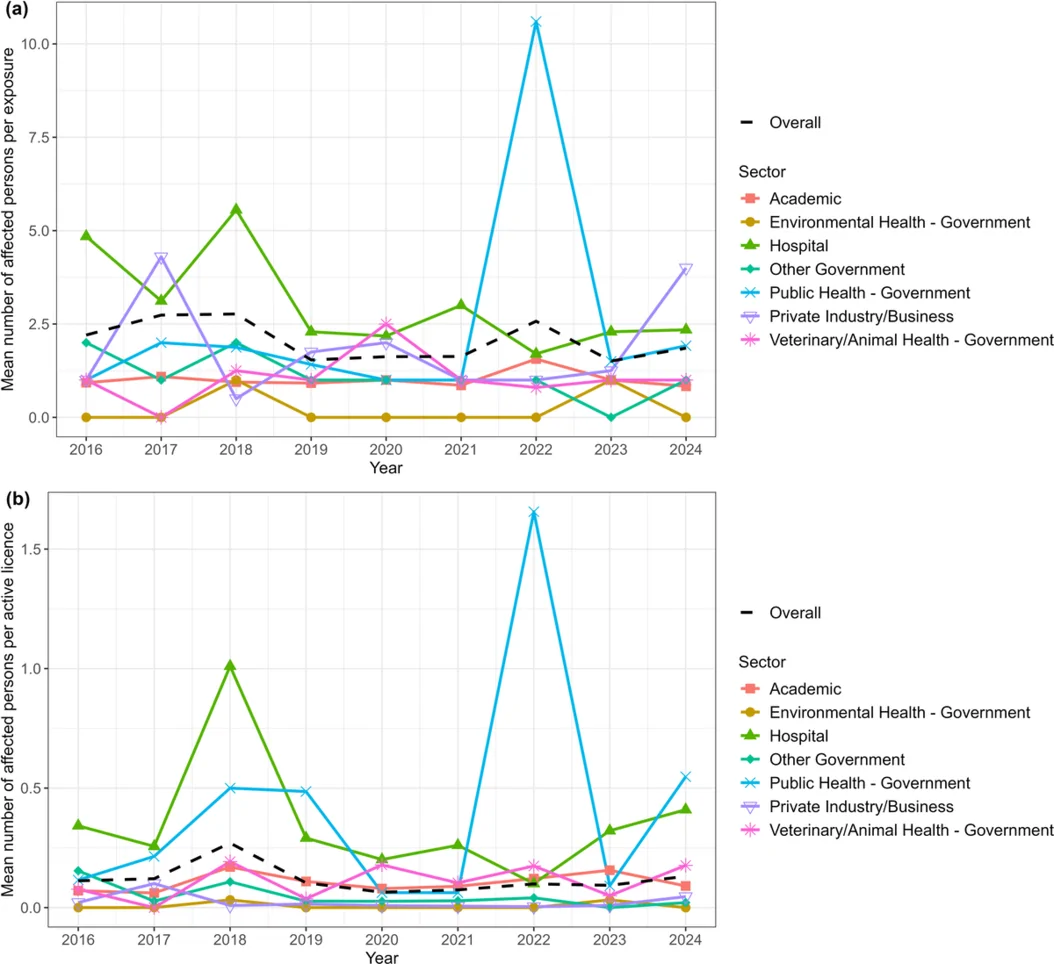
Mean number of affected persons per (a) exposure incident and (b) active licence by sector and overall, 2016–2024. The proportion is represented on the y‐axis and the year is represented on the x‐axis. Each sector is represented by a different color and the overall trend is represented by a dashed, black line.

The mean number of APs per exposure incident decreased from 2016 until 2022, with a slight increase observed since 2023. However, a linear trend analysis did not identify a statistically significant temporal trend (*p* = 0.21). The mean number of APs per exposure incident in the other government sector followed a similar pattern, though the increase began in 2024. In the private industry/business sector, the mean number of APs per exposure incident fluctuated throughout the years, while the mean number of APs per active licence remained consistent. The most notable increase was observed in the public health sector in 2022, with an average of 10.6 APs per exposure and 1.66 APs per active licence. Throughout the years, the hospital and public health sectors had the highest mean number of APs per exposure incident (mean ± SD = 3.04 ± 1.31 and mean ± SD = 2.48 ± 3.07, respectively) and per active licence (mean ± SD = 0.35 ± 0.26 and mean ± SD = 0.42 ± 0.51, respectively), while the environmental health had the lowest mean number of APs per exposure (mean ± SD = 0.22 ± 0.44) and per active licence (mean ± SD = 0.01 ± 0.01).

## 4 Discussion

This study analyzed 1058 individuals affected in confirmed exposure incidents in licensed facilities in Canada from 2016 to 2024. The majority of APs were in the non‐LAI group (96.6%). APs in both the LAI group and the non‐LAI group were predominantly technicians/technologists or students, held a technical/trades college diploma or bachelor's degree as their highest education level, and were performing microbiology activities at the time of exposure. Similar findings have been reported in previous studies involving LINC data [1, 2, 3, 17].

Statistically significant differences between APs in the LAI group and the non‐LAI group were observed for multiple AP‐related variables, emphasizing the importance of studying APs from both disease outcomes. Though most of the literature on APs are exclusive to those affected in LAI‐associated incidents [5, 9], this study, which included APs in LAI‐associated incidents and non‐LAI incidents, found that APs in the LAI group were generally less experienced, were more likely to work in the academic sector, and had higher levels of education, compared to APs in the non‐LAI group. While this observation may suggest a potential role for experience shaping disease outcomes, the effect size was negligible and the association was not supported in the exploratory regression analyses. Additional studies with larger numbers of APs in the LAI group are needed to determine whether years of experience is independently associated with the development of an LAI. As observed in this study, APs with a technical/trades diploma, which involve a shorter period of formal education compared to a university degree, had the highest mean and median years of laboratory experience and are more likely to be in the non‐LAI group. Generally, APs in the non‐LAI group had more years of laboratory experience, were technicians/technologists, and were working in the hospital sector. In both the LAI and non‐LAI groups, the predominant routes of exposure to HPTs for APs were through facial orifices (i.e., via ingestion or inhalation), thus underscoring the importance of wearing facial PPE during laboratory activities to prevent exposure incidents. While glove use was generally high, laboratory personnel have been observed to be less compliant with other forms of PPE, particularly with use of laboratory coats and eye protection, with notable differences across institutional sectors [19]. While certain factors differed between those affected in the LAI group and the non‐LAI group, the practical magnitude of these differences was limited. Effect‐size measures were generally small or negligible, indicating that statistically significant differences did not necessarily translate into meaningful practical differences. In an exploratory analysis, Firth's penalized logistic regression was used to assess whether selected AP characteristics and exposure‐related factors were associated with the development of an LAI and to identify potential areas for future hypothesis‐driven research. Moderate exposure incident severity was associated with increased odds of the development of an LAI. However, these findings should be interpreted in the context of the small number of APs in the LAI group reported during the study period and sparse subgroup counts for several variables. Many estimates were accompanied by wide confidence intervals, indicating limited precision. As a result, the analysis was considered hypothesis‐generating rather than confirmatory. Future studies with larger numbers of APs in the LAI group may help clarify whether exposure severity and other AP characteristics are associated with the development of an LAI.

One of the most notable differences between APs in the LAI group and the non‐LAI group was the higher prevalence of the “unknown” category for the route of exposure, exposure severity, and root causes among APs in the LAI group. While this cannot be classified as missing data, it can be considered non‐informative, introducing ambiguity and challenges in data interpretation. Several factors could explain this lack of information, such as the high‐stress nature of an LAI‐associated incident, or the time delay between the occurrence and symptom onset or incident reporting, leading to difficulties in memory retrieval of the incident [20, 21]. Though information on the number of days between exposure and symptom onset is not collected by LINC, APs in the LAI group were less likely to seek certain post‐exposure interventions within seven days of the incident and more likely to seek certain interventions more than seven days after the incident. This suggests a potential time delay between the incident date and onset or reporting date, which could reduce the AP's ability to recall details of the LAI‐associated incident [21].

Of the seven sectors that were included in the sector‐specific analyses, the academic and veterinary/animal health sectors revealed the most variability in the distribution of APs for multiple AP‐related variables, namely role, highest education level, route of exposure, and main activity during exposure. The variability in the academic sector might be attributable to the diversity of roles of the laboratory personnel, which is expected because of the teaching environment in academic institutions. In the veterinary/animal health sector, variability among the APs may also stem from the broad range of roles, education levels, or tasks (e.g. animal blood/tissue collection, necropsy, histopathology, etc.) of personnel. In other sectors, such as the hospital, public health, and private/business sectors, APs were predominantly affected through procedure‐related occurrences and may potentially present less variability because the facilities in these sectors tend to perform more repetitive, standardized laboratory tests. As for APs in the veterinary/animal health sector, the higher frequency of sharps‐related exposures aligned with previous findings that injury from needlesticks was a common occurrence amongst veterinary technicians with time constraints, improper handling of needles by veterinarians, and inadequate assistance during animal restraint as the leading contributing factors [22]. These results highlight the importance of having a strong prevention education on sharps injuries, evaluating risks, and continuously improving the quality of procedures to prevent adverse events.

The strength of safety culture and perception in facilities may be suggested by the number of post‐exposure interventions implemented. Given that APs in the public health and private industry/business sectors received the most post‐exposure interventions per AP on average, more emphasis on safety culture and safety perception may be present in these sectors. Conversely, the lower number of post‐exposure interventions per AP in the veterinary/animal health and academic sectors may suggest potential areas to enhance and put emphasis on safety culture and safety perception. However, differences in post‐exposure interventions across sectors may also reflect variation in activities conducted in facilities, hazards encountered, biological agents involved, institutional policies, risk assessment outcomes, and access to occupational health and medical resources. The reporting and implementation of post‐exposure interventions may also depend on the AP's willingness to seek medical attention, share information, and adhere to the recommended interventions. Therefore, differences in the number of reported post‐exposure interventions may not solely be interpreted as differences in organizational safety culture, as they may also be influenced by individual behaviors and the nature and severity of exposure incidents themselves.

The difference in safety culture and perception as observed through PPE compliance behavior across the sectors has been explored in other studies. For instance, one study found that laboratory personnel in the government and industry sectors wore laboratory coats and eye protection more frequently than those in the academic sector [19]. Similarly, the proportions of APs involved in PPE‐related occurrences in the private industry/business and public health sectors (3.0%–8.9%) in this study were lower than in the veterinary/animal health and academic sectors (24.9%–29.2%). Further, a study examining laboratory animal workers’ perception of occupational risk and injury noted that while workers believed it was important to wear recommended PPE, many did not believe that more consistent use of PPE would reduce the risk of infection or injury, nor that hand washing is important prior to removing facial PPE [23].

The most common HPTs involved in all exposure incidents were *Brucella* species and were most often reported in the academic, hospital, public health, and private industry/business sectors. *Brucella* isolates are known to be transmissible through ingestion, inhalation, and direct contact with skin via abrasions and mucous membranes [24]. One of the most common sources of laboratory‐acquired brucellosis is exposure to *Brucella* species during the identification of isolates from blood specimens [25]. These agents have commonly been implicated in LAI‐associated incidents [7, 24]. With clinical microbiology personnel being at increased risk of occupational infection due to the presence of potentially pathogenic organisms in clinical specimens, the consistent use of biological safety cabinets may reduce exposure risk and LAIs [25]. The mean number of APs per exposure incident decreased over the study period; however, this trend was not statistically significant. The observed decline, therefore, should be interpreted cautiously and may reflect year‐to‐year variability in reported incidents, activities conducted in facilities, or reporting practices. Nevertheless, efforts to strengthen biosafety measures may help limit the number of individuals affected when exposure incidents occur.

There were some limitations in this study. When submitting a report to PHAC, licensed facilities are not required to disclose demographic or workforce information. Consequently, denominator data describing the workforce characteristics within licensed facilities were unavailable. Thus, analyses comparing APs and unaffected individuals in a facility could not be conducted. As a result, proportions in this study were only relative to the total APs reported to PHAC and should not be interpreted as indicators of occupational risk, overrepresentation, or susceptibility. Rather, these findings provide a descriptive summary of reported APs and when interpreting results related to the characteristics of APs, it should be noted that corresponding distributions in the underlying workforce are unknown, so a comparison is not possible. In addition, only 36 APs in the LAI group were reported during the study period (compared to 1022 APs in the non‐LAI group), and only three APs were involved in incidents classified as of major severity. Multiple APs may be associated with an incident, and exposure severity is based on subjective facility‐reported assessments. An exploratory Firth's penalized logistic regression analysis identified a potential association between moderate exposure incident severity and the development of an LAI; however, several estimates of other characteristics had wide confidence intervals, reflecting sparse data and limited precision. As such, the findings should be considered hypothesis‐generating, and the ability to identify independent predictors of LAI‐associated incidents or more severe exposure outcomes remained limited.

Another limitation is the imbalanced sample sizes between APs in the LAI group and the non‐LAI group, and across sectors. Small subgroup sizes may yield reduced statistical power, increased uncertainty around estimates, and limited reliability of subgroup comparison. As such, any statistically significant findings from this study should be considered in the context of these sample size limitations. Particularly, observations concerning APs in the environmental health sector cannot be considered as meaningful sector‐specific patterns due to the extremely small sample size (*n* = 2), as well as those in the veterinary/animal health sector (*n* = 24). Further, given that surveillance data are self‐reported, there is the possibility of recall bias. Finally, while reporting exposure incidents is mandatory, under‐reporting may still occur. The extent of under‐reporting, especially by sector, cannot be ascertained.

## 5 Conclusion

Most individuals affected in exposure incidents in licensed facilities in Canada from 2016 to 2024 were involved in non‐LAI incidents. Many characteristics of APs (i.e., highest education level, years of laboratory experience, sector, route of exposure, main activity during exposure, exposure severity) differed by disease outcome (LAI group and non‐LAI group), with APs in the LAI group generally more likely to implement post‐exposure interventions more than seven days after exposure. The characteristics of APs varied most greatly in the academic, other government, and veterinary/animal health sectors, and they exhibited the least heterogeneity in the hospital and public health sectors. Species of the *Brucella* genus were among the most commonly implicated agents across all sectors. Despite some fluctuations, the mean number of APs per exposure and per active licence throughout the years remained relatively consistent with a slight decreasing trend in the mean number of APs per exposure.

By analyzing variables pertaining to the persons affected in exposure incidents in Canada, this study contributes to the descriptive characterization of the educational, occupational, and exposure‐related profiles of reported APs. These findings provide context for licensed facilities’ biosafety programs and may help guide future research aimed at identifying factors associated with exposure incidents and inform evidence‐based prevention strategies.

## Author Contributions

Conceptualization, methodology, data analysis and interpretation: Emily F. Tran and Christine Abalos; Data validation: Emily F. Tran and Christine Abalos; Writing – original draft: Emily F. Tran and Christine Abalos; Writing – review and editing: Emily F. Tran, Christine Abalos, Antoinette Davis, Audrey Gauthier and Samuel Bonti‐Ankomah; Supervision: Antoinette N. Davis.

## Ethics Statement

This study was conducted at the Public Health Agency of Canada. The Public Health Agency of Canada determined that this project was exempt from ethics review because it involved analysis of public health surveillance data. Informed consent was not required.

## Supporting information


Supporting File 1

Supporting File 2

Supporting File 3

Supporting File 4

Supporting File 5

Supporting File 6

## Data Availability

Research data are not shared. The data that support the findings of this study are not publicly available due to privacy restrictions. Select exposure, non‐exposure and other reporting data from licensed facilities in Canada from 2025 onwards can be found on Open Government https://open.canada.ca/data/en/dataset/c2b10948-097d-4cbf-ab07-8581a77a95d5.
