# Supplementary material for: Affected Persons in Laboratory Exposures to Human Pathogens and Toxins in Canada, 2016–2024: A Sector‐Specific Analysis
Source: Am J Ind Med. 2026 Sep 15;69(12):1080–92. doi: 10.1002/ajim.70137 (PMC13740951; doi:10.1002/ajim.70137)
Supplement: Supplementary file 1 [file AJIM-69-1080-s001.docx]

**Table SIV** Root causes and examples of areas of concern

| Root cause | Examples of areas of concern |
| --- | --- |
| Human factors | A violation (cutting a corner, not follow correct procedure, deviating from standard operating procedure) |
|  | An error (a mistake, lapse of concentration, or slip of any kind) |
| SOP | Documents were followed as written but not correct for activity/task |
|  | Procedures that should have been in place were not in place |
|  | Documents were not followed correctly |
| Training | Training was not in place but should have been in place |
|  | Training was not appropriate for the task/activity |
|  | Staff were not qualified or proficient in performing task |
| Management and oversight | Supervision needed improvement |
|  | Lack of auditing of standards, policies and procedures |
|  | Risk assessment needed improvement |
| Equipment | Equipment quality control needed improvement |
|  | Equipment failed |
|  | Equipment was not appropriate for purpose |
| Communication | Communication did not occur but should have |
|  | Communication was unclear, ambiguous, etc. |
| Other | Root causes not captured elsewhere |
|  | Unpredictable/random movement by research animal |

Abbreviation: SOP (standard operating procedure)
