# Supplementary material for: Affected Persons in Laboratory Exposures to Human Pathogens and Toxins in Canada, 2016–2024: A Sector‐Specific Analysis
Source: Am J Ind Med. 2026 Sep 15;69(12):1080–92. doi: 10.1002/ajim.70137 (PMC13740951; doi:10.1002/ajim.70137)
Supplement: Supplementary file 2 [file AJIM-69-1080-s002.docx]

**Table SV** Distribution of affected persons among occurrence types by disease outcome (LAI group vs. non-LAI group), 2016–2024 (N=1,058)

|  | LAI group  (n=36; 3.4%) | | Non-LAI group  (n=1,022; 96.6%) | | Total  (N=1,058) | | P-value |
| --- | --- | --- | --- | --- | --- | --- | --- |
|  | n | % | n | % | n | % |  |
| OT–Animal |  |  |  |  |  |  | 0.311 |
| Yes | 2 | 5.6 | 31 | 3.0 | 33 | 3.1 |  |
| No | 34 | 94.4 | 991 | 97.0 | 1025 | 96.9 |  |
| OT–Equipment |  |  |  |  |  |  | 0.253 |
| Yes | 0 | 0 | 55 | 5.4 | 55 | 5.2 |  |
| No | 36 | 100 | 967 | 94.6 | 1003 | 94.8 |  |
| OT–Insect |  |  |  |  |  |  | 1.00 |
| Yes | 0 | 0 | 3 | 0.3 | 3 | 0.3 |  |
| No | 36 | 100 | 1019 | 99.7 | 1055 | 99.7 |  |
| OT–Loss of containment |  |  |  |  |  |  | 0.251 |
| Yes | 1 | 2.8 | 105 | 10.3 | 106 | 10.0 |  |
| No | 35 | 97.2 | 917 | 89.7 | 952 | 90.0 |  |
| OT–Other |  |  |  |  |  |  | 0.074 |
| Yes | 4 | 11.1 | 250 | 24.5 | 254 | 24.0 |  |
| No | 32 | 88.9 | 772 | 75.5 | 804 | 76.0 |  |
| OT–PPE |  |  |  |  |  |  | 0.271 |
| Yes | 9 | 25.0 | 182 | 17.8 | 191 | 18.1 |  |
| No | 27 | 75.0 | 840 | 82.2 | 867 | 81.9 |  |
| OT–Procedure |  |  |  |  |  |  | 0.003 |
| Yes | 9 | 25.0 | 514 | 50.3 | 523 | 49.4 |  |
| No | 27 | 75.0 | 508 | 49.7 | 535 | 50.6 |  |
| OT–Sharps |  |  |  |  |  |  | 0.447 |
| Yes | 6 | 16.7 | 129 | 12.6 | 135 | 12.8 |  |
| No | 30 | 83.3 | 893 | 87.4 | 923 | 87.2 |  |
| OT–Spill |  |  |  |  |  |  | 0.244 |
| Yes | 1 | 2.8 | 96 | 9.4 | 97 | 9.2 |  |
| No | 35 | 97.2 | 926 | 90.6 | 961 | 90.8 |  |
| OT–Unknown |  |  |  |  |  |  | <0.001 |
| Yes | 17 | 47.2 | 18 | 1.8 | 35 | 3.3 |  |
| No | 19 | 52.8 | 1004 | 98.2 | 1023 | 96.7 |  |

Abbreviations: OT (occurrence type); PPE (personal protective equipment)
