# Supplementary material for: Affected Persons in Laboratory Exposures to Human Pathogens and Toxins in Canada, 2016–2024: A Sector‐Specific Analysis
Source: Am J Ind Med. 2026 Sep 15;69(12):1080–92. doi: 10.1002/ajim.70137 (PMC13740951; doi:10.1002/ajim.70137)
Supplement: Supplementary file 3 [file AJIM-69-1080-s003.docx]

**Table SII** Definitions of occurrence types

| Occurrence type | Definition |
| --- | --- |
| Spill | Any unintended release of an agent from its container |
| Loss of containment | Includes malfunction or misuse of containment devices or equipment and other type of failures that results in the agent being spilled outside of, or released from, containment |
| Sharps-related | Includes needle stick, cut with scalpel, blade or other sharps injury (i.e., broken glass) |
| Animal-related | Includes animal bites or scratches, as well as other exposure incidents resulting from animal behaviour (i.e., animal movement resulting in a needle stick) |
| Insect-related | Includes insect bites |
| PPE-related | Includes either inadequate PPE for the activity or failure of the PPE in some way |
| Equipment-related | Includes failure of equipment, incorrect equipment for the activity or misuse of equipment |
| Procedure-related | Includes instances when written procedures were not followed, were incorrect for the activity or were inadequate or absent |
| Other | Includes occurrence types that did not belong to any of the above |
| Unknown | Includes occurrence types that were inconclusive |

Abbreviations: PPE (personal protective equipment)
