# Supplementary material for: Affected Persons in Laboratory Exposures to Human Pathogens and Toxins in Canada, 2016–2024: A Sector‐Specific Analysis
Source: Am J Ind Med. 2026 Sep 15;69(12):1080–92. doi: 10.1002/ajim.70137 (PMC13740951; doi:10.1002/ajim.70137)
Supplement: Supplementary file 4 [file AJIM-69-1080-s004.docx]

**Table SI** Definitions of main activities

| Main activity | Definition |
| --- | --- |
| Animal care | Activities such as attending to the daily care of animals and providing animals with treatment |
| Autopsy or necropsy | Post-mortem surgical examinations for purposes such as determining cause of death or to evaluate disease or injury for research or educational purposes |
| Cell culture | The process of growing cells under controlled conditions—it can also involve the removal of cells from an animal or plant |
| Education or training | Education or training of students and/or personnel on laboratory techniques and procedures |
| *In vivo* animal research | Experimentation with live, non-human animals |
| Maintenance | The upkeep, repair and/or routine and general cleaning of equipment and facilities |
| Microbiology | Activities involving the manipulation, isolation or analysis of microorganisms in their viable or infectious state |
| Molecular investigations | Activities involving the manipulation of genetic material from microorganisms or other infectious material for further analysis |
| Serology | Diagnostic examination and/or scientific study of immunological reactions and properties of blood serum |
| Hematology | Scientific study of the physiology of blood |
| Other | Other type of activity not captured elsewhere |
