# Supplementary material for: Affected Persons in Laboratory Exposures to Human Pathogens and Toxins in Canada, 2016–2024: A Sector‐Specific Analysis
Source: Am J Ind Med. 2026 Sep 15;69(12):1080–92. doi: 10.1002/ajim.70137 (PMC13740951; doi:10.1002/ajim.70137)
Supplement: Supplementary file 5 [file AJIM-69-1080-s005.docx]

**Table SIII** Definitions of exposure severities

| Exposure severity | Definition |
| --- | --- |
| Negligible | Minimal risk of disease in the individual/other staff and no risk to public health |
| Minor | Low risk of disease in the individual/other staff and/or low risk to public health |
| Moderate | Moderate risk of disease in the individual/other staff and/or moderate risk to public health (limited spread among close contacts, no deaths) |
| Major | High risk of severe disease/death in the individual/other staff and/or significant public health impact (community spread/outbreak/fatalities) |
