# Supplementary material for: Affected Persons in Laboratory Exposures to Human Pathogens and Toxins in Canada, 2016–2024: A Sector‐Specific Analysis
Source: Am J Ind Med. 2026 Sep 15;69(12):1080–92. doi: 10.1002/ajim.70137 (PMC13740951; doi:10.1002/ajim.70137)
Supplement: Supplementary file 6 [file AJIM-69-1080-s006.docx]

**Table SVI** Distribution of affected persons among root causes of exposures by disease outcome (LAI group vs. non-LAI group), 2016–2024 (N=1,058)

|  | LAI group  (n=36; 3.4%) | | Non-LAI group  (n=1,022; 96.6%) | | Total  (N=1,058) | | P-value |
| --- | --- | --- | --- | --- | --- | --- | --- |
|  | n | % | n | % | n | % |  |
| RC–Training |  |  |  |  |  |  | 0.005 |
| Yes | 8 | 22.2 | 335 | 32.8 | 343 | 32.4 |  |
| No | 22 | 61.1 | 640 | 62.6 | 662 | 62.6 |  |
| Not applicable | 1 | 2.8 | 10 | 1.0 | 11 | 1.0 |  |
| Unknown | 5 | 13.9 | 21 | 2.1 | 26 | 2.5 |  |
| Missing | 0 | 0 | 16 | 1.6 | 16 | 1.5 |  |
| RC–Communication |  |  |  |  |  |  | 0.202 |
| Yes | 7 | 19.4 | 324 | 31.7 | 331 | 31.3 |  |
| No | 27 | 75.0 | 654 | 64.0 | 681 | 64.4 |  |
| Not applicable | 1 | 2.8 | 10 | 1.0 | 11 | 1.0 |  |
| Unknown | 1 | 2.8 | 15 | 1.5 | 16 | 1.5 |  |
| Missing | 0 | 0 | 19 | 1.9 | 19 | 1.8 |  |
| RC–Equipment |  |  |  |  |  |  | <0.001 |
| Yes | 8 | 22.2 | 252 | 24.7 | 260 | 24.6 |  |
| No | 25 | 69.4 | 748 | 73.2 | 773 | 73.1 |  |
| Not applicable | 0 | 0 | 6 | 0.6 | 6 | 0.6 |  |
| Unknown | 3 | 8.3 | 0 | 0 | 3 | 0.3 |  |
| Missing | 0 | 0 | 16 | 1.6 | 16 | 1.5 |  |
| RC–Human factors |  |  |  |  |  |  | <0.001 |
| Yes | 17 | 47.2 | 540 | 52.8 | 557 | 52.6 |  |
| No | 12 | 33.3 | 447 | 43.7 | 459 | 43.4 |  |
| Not applicable | 1 | 2.8 | 8 | 0.8 | 9 | 0.9 |  |
| Unknown | 6 | 16.7 | 12 | 1.2 | 18 | 1.7 |  |
| Missing | 0 | 0 | 15 | 1.5 | 15 | 1.4 |  |
| RC–Management |  |  |  |  |  |  | 0.002 |
| Yes | 8 | 22.2 | 243 | 23.8 | 251 | 23.7 |  |
| No | 24 | 66.7 | 750 | 73.4 | 774 | 73.2 |  |
| Not applicable | 1 | 2.8 | 10 | 1.0 | 11 | 1.0 |  |
| Unknown | 3 | 8.3 | 3 | 0.3 | 6 | 0.6 |  |
| Missing | 0 | 0 | 16 | 1.6 | 16 | 1.5 |  |
| RC–Other |  |  |  |  |  |  | 0.186 |
| Yes | 10 | 27.8 | 186 | 18.2 | 196 | 18.5 |  |
| No | 26 | 72.2 | 836 | 81.8 | 862 | 81.5 |  |
| RC–SOP |  |  |  |  |  |  | 0.030 |
| Yes | 18 | 50.0 | 615 | 60.2 | 633 | 59.8 |  |
| No | 12 | 33.3 | 348 | 34.1 | 360 | 34.0 |  |
| Not applicable | 2 | 5.6 | 14 | 1.4 | 16 | 1.5 |  |
| Unknown | 4 | 11.1 | 30 | 2.9 | 34 | 3.2 |  |
| Missing | 0 | 0 | 15 | 1.5 | 15 | 1.4 |  |

Note: Due to rounding of percentage values, the sum of individual percentage values may exceed 100% by 0.1%.

Abbreviations: RC (root cause); SOP (standard operating procedure)
